# Towards ensuring reproducibility of outsourced data generation

**DOI:** 10.1371/journal.pbio.3002988

**Published:** 2025-01-15

**Authors:** Daniel B. Sloan, Mark D. Stenglein

**Affiliations:** 1 Department of Biology, Colorado State University, Fort Collins, Colorado, United States of America; 2 Department of Microbiology, Immunology, and Pathology, Colorado State University, Fort Collins, Colorado, United States of America

## Abstract

‘Big data’ generated from outsourced or centralized facilities often lacks methodological information. This Perspective outlines how and why researchers, service providers, and other parties should report on methodology and sample metadata to improve scientific reproducibility.

## Teaser

“Big data” generated from outsourced or centralized facilities often lacks methodological information. Here, we outline how and why researchers, service providers, and other parties should report on methodology and sample metadata to improve scientific reproducibility.

Nearly every corner of the sciences has been transformed by technologies that produce massive data sets, placing us in the era of “big data” [1]. This progress has been accelerated by specialized companies and core facilities that can provide much greater throughput and efficiency than individual research labs. For the purposes of this Perspective, we focus on DNA sequencing facilities, but the general points pertain to centralized data collection in areas such as proteomics, metabolomics, cryo-electron microscopy and structural biology, high-throughput screening and drug discovery, and isotope/chemical analyses.

Facilities that generate data at scale are equipped with cutting-edge scientific instrumentation and staffed by experts with extensive scientific training. However, the day-to-day mission of these facilities is more akin to the manufacturing industry than to research science. Facilities are contracted to generate a product (for instance, DNA sequence data) and are part of a crowded market with significant competition over pricing. Commercial enterprises face profit expectations, and university core facilities operate under substantial budgetary constraints, creating incentives that are sometimes at odds with the maximization of scientific rigor and reproducibility. Communicating detailed protocol information to customers comes at a cost to other facility operations. Therefore, it is not surprising that the default mode of operation for many sequencing facilities (in our experience) is to return data with little or no methods documentation. As such, the scientists involved in the project and the research community at large risk losing track of key information.

As data generation becomes increasingly centralized and commoditized, we anticipate that the problem will worsen, with researchers being further removed from the production process and more accustomed to receiving data from fee-for-service contractual exchanges. Although it is commonplace for researchers to purchase reagents from commercial providers and simply reference the source without information about how the reagents were produced, we contend that treating sequencing facilities in a similar black-box fashion is problematic. The high-throughput technologies that are driving progress in genomics and other big-data fields are evolving too fast. There are numerous cases where identification of sequencing artefacts has overturned initial scientific conclusions and raised questions about whether other studies suffer from the same methodological problems [2–6]. It is especially important to record methodological information when facilities process samples and construct sequencing libraries, activities that are more complex than data generation from existing libraries. Mundane details such as the DNA polymerase and number of PCR cycles used for library amplification can determine the amount of bias in sequence representation [7]. More generally, even in the absence of any technical errors or artefacts, proper (re)interpretation of sequencing data sets can depend on the specifics of how they were produced [8–10]. For example, whether reads represent the coding or template strand (or a mix of both) in RNA-seq data sets depends on the methods used during library prep [11].

One challenge arising from centralized data generation is that researchers often lack direct experience with the methods and, thus, may not know what questions to ask. As such, obtaining key methodological information may require back-and-forth exchanges among researchers, facility personnel, and other experts in the field. Early efforts were made to establish standardized reporting requirements for high-throughput sequencing experiments, including both wet-lab and computational methods [12,13]. However, these requirements have never reached widespread adoption in the same way that, for example, the Minimum Information for Publication of Quantitative Real-Time PCR Experiments (MIQE) guidelines defined norms for reporting experimental methods for quantitative PCR projects [14]. In addition, requirements for submission to repositories such as the NCBI Sequence Read Archive (SRA) have often placed greater emphasis on (very important) metadata about biological samples than on methods for generation of the sequence data itself.

Although the ultimate responsibility for recording and reporting methodological information lies with researchers, all parties can contribute to ensuring reproducibility in outsourced data generation (Box 1). Simple steps that researchers can take include confirming from the onset of a project that sequencing facilities will provide protocol information and making sure they do so upon project completion. In our experience, facilities are usually happy to supply this information (with rare exceptions raising a big red flag), but it generally requires a proactive request. Researchers should establish lab policies to record those methods and report them in publications and data depositions, just as they would for work conducted in their own labs. Peer reviewers, publishers, and funders also have roles to play (Box 1) by insisting on full methods reporting during manuscript review and as a condition of funding. Data repositories like the NCBI SRA could enforce these policies by requiring that data submissions be paired with more complete methodological information.

## Box 1 –Steps towards ensuring reproducibility in outsourced data generation

**Researchers**

- When first contracting with service providers, confirm that methodological information will be made available.
- Verify that complete methodological information is provided upon data delivery and follow up with clarifying methods questions in a timely fashion.
- Preferentially work with service providers with strong track records of reporting detailed methods and cease doing business with those that fail to provide this information.
- Report methodological details from external service providers in papers and when depositing data.
- Establish laboratory guidelines that reinforce these practices with junior trainees.

**Service Providers**

- Generate protocols or standard operating procedures (SOPs) that contain sufficient detail to ensure reproducibility and that can be shared with customers.
- Record methodological metadata (protocol/chemistry/software versions, instrument models, etc.) that can be associated with each round of data generation.
- Deliver methodological information automatically with data (not just upon request).

**Funders**

- Require plans for obtaining and reporting methodological information from outsourced data generators as part of data management and dissemination plans.
- Prohibit expenditures of funds on service providers that fail to report methodological information.

**Journals/Editors**

- Instruct authors and peer reviewers that standard expectations for complete methods reporting also apply to outsourced data generation.
- Enforce expectations for complete methods requirements as a criterion for final publication.

**Peer Reviewers**

- Flag grant proposals that do not show adequate plans to obtain, store, and report methodological information from outsourced data generators.
- Insist that manuscripts include sufficient methodological detail for externally generated data.

The challenges outlined in this Perspective stem from the centralization of data generation in the hands of core facilities and commercial providers. Fortunately, this centralization also presents an opportunity because it should be more manageable to standardize methods reporting through coordination with a relatively small number of data generators than through the actions of all research labs individually. We envision that a community effort can create incentives that align the interests of researchers and data-generating “manufacturers” and make it standard practice for methods reporting to accompany data delivery.
